# Non-parametric estimation of reference adjusted, standardised probabilities of all-cause death and death due to cancer for population group comparisons

**DOI:** 10.1186/s12874-021-01465-w

**Published:** 2022-01-06

**Authors:** Mark J. Rutherford, Therese M.-L. Andersson, Tor Åge Myklebust, Bjørn Møller, Paul C. Lambert

**Affiliations:** 1 Department of Health Sciences, University of Leicester, Leicester, UK; 2 Medical Epidemiology and Biostatistics, Karolinska Institutet, Stockholm, Sweden; 3 Department of Registration, Cancer Registry of Norway, Oslo, Norway; 4 Department of Research and Innovation, Møre and Romsdal Hospital Trust, Ålesund, Norway

**Keywords:** Age-standardisation, Net survival, Crude probability of death, Competing risks

## Abstract

**Background:**

Ensuring fair comparisons of cancer survival statistics across population groups requires careful consideration of differential competing mortality due to other causes, and adjusting for imbalances over groups in other prognostic covariates (e.g. age). This has typically been achieved using comparisons of age-standardised net survival, with age standardisation addressing covariate imbalance, and the net estimates removing differences in competing mortality from other causes. However, these estimates lack ease of interpretability. In this paper, we motivate an alternative non-parametric approach that uses a common rate of other cause mortality across groups to give reference-adjusted estimates of the all-cause and cause-specific crude probability of death in contrast to solely reporting net survival estimates.

**Methods:**

We develop the methodology for a non-parametric equivalent of standardised and reference adjusted crude probabilities of death, building on the estimation of non-parametric crude probabilities of death. We illustrate the approach using regional comparisons of survival following a diagnosis of rectal cancer for men in England. We standardise to the covariate distribution and other cause mortality of England as a whole to offer comparability, but with close approximation to the observed all-cause region-specific mortality.

**Results:**

The approach gives comparable estimates to observed crude probabilities of death, but allows direct comparison across population groups with different covariate profiles and competing mortality patterns. In our illustrative example, we show that regional variations in survival following a diagnosis of rectal cancer persist even after accounting for the variation in deprivation, age at diagnosis and other cause mortality.

**Conclusions:**

The methodological approach of using standardised and reference adjusted metrics offers an appealing approach for future cancer survival comparison studies and routinely published cancer statistics. Our non-parametric estimation approach through the use of weighting offers the ability to estimate comparable survival estimates without the need for statistical modelling.

**Supplementary Information:**

The online version contains supplementary material available at 10.1186/s12874-021-01465-w.

## Background

Net survival measures are typically used for population-based cancer data as they enable fair comparisons across population groups which have differential competing risks due to deaths from causes other than cancer [1, 2]. Past comparisons of individuals diagnosed with a specific cancer have been made between groups defined by geographical areas [3–6], calendar time [7] or by population subgroupings; such as age [8], socioeconomic status [9] or race [10]. In the context of cancer survival, net survival measures the survival in the hypothetical world where it is not possible to die from causes other than the cancer of interest. However, net survival measures have been criticized as lacking a directly relevant interpretation, with some cautioning against relying on metrics that do not “stick to this world” [11]. A further consideration when comparing population subgroups is that care should be taken to ensure that the observed covariate distribution (age is the one primarily considered) in each group is similar, or are enforced through some form of weighting or standardization. See, for example, Corazziari et al. [12] for accounting for age distribution differences. The same approach can be applied for other key covariates, dependent on the cancer site and question of interest.

Crude probability measures (also referred to as cause-specific cumulative incidence functions in the competing risks literature) offer a more interpretable metric, and have the advantage of being a real-world measure. The cancer-specific crude probability of death measures the risk of dying of a cancer at a particular timepoint in the presence of the competing risks due to other causes of death. However, crude probabilities are a function of both the cancer-specific (or excess) mortality rate and the other cause mortality rate, and so comparison between population groups are not “fair” when trying to isolate differences solely due to the impact of cancer. This lack of “fairness” motivates the use of net survival in the first place, rather than relying on the all-cause survival across groups, which is also impacted by both competing mortality rates. Crude probability metrics have received some recent attention in the relative survival framework, with various estimation approaches proposed [13–16] and their use in a number of applied contexts [17–22].

Lambert et al. [23] propose estimation of all-cause survival and crude probability measures where differences between population groups only depend on differences in excess (cancer) mortality rates; they use the terminology of reference adjusted measures. In order to estimate the reference-adjusted crude probability and all-cause measures in a relative survival (excess mortality) framework, Lambert et al. [23] propose using common other-cause mortality rates from a reference population when converting back from the excess mortality scale (which may well be the other-cause mortality rates from one of the groups of interest). This leads to metrics that may not reflect the actual experience of a population group, but are comparable and offer improved interpretability. With careful selection of the appropriate reference adjustment and standardisation, these metrics can also closely reflect the real-world experience of the study population. It is important to note that the relevant other cause mortality rate for each group are first used in the estimation of the excess mortality rates, prior to using the common other cause mortality rates for all groups when converting to the all-cause scale.

In this paper, we build on the ideas of Lambert et al. [23], through development of non-parametric methods to estimate the same underlying estimands. The approach of Lambert et al. relies on a fully parametric setting; requiring the correct specification of the functional form for non-linear and time-dependent covariate effects in a modelling framework. Our proposed non-parametric alternative removes the requirement of the correct model specification. We apply the developed non-parametric estimators and explore regional differences in survival following a diagnosis of rectal cancer in England. This approach also builds upon previous research from Cronin and Feuer [24] for estimating crude probability metrics, but we instead apply reference population other-cause mortality rates to ensure that the crude probability metrics are fair in terms of differential other-cause mortality when comparing across population groups. In terms of implementation, we discuss the calculation of the metrics in both continuous time (that is, at unique event times), and with follow-up time grouped into intervals.

## Methods

### Statistical methods

We develop our estimation approaches within a relative survival (excess mortality) framework. This is the most common approach to survival estimation in population-based cancer data, largely because of the unavailability or unreliability of dichotomising cause of death information into a death either due to cancer or due to other causes, which would be required in a cause-specific survival estimation framework. We start by considering the all-cause mortality rate, *h*_*i*_(*t*), for an individual *i* at time from diagnosis, *t*, which is assumed can be partitioned into component parts; that due to the background mortality rate, ${h}_i^{\ast }(t),$ typically defined by information from population mortality files and the excess mortality rate, *λ*_*i*_(*t*), for mortality associated with the diagnosis of cancer:

$${h}_i\left(\mathrm{t}\right)={h}_i^{\ast }(t)+{\lambda}_i(t).$$

The subscript *i* denotes that this partitioning will vary by individual patient characteristics that could impact the background mortality, the cancer-specific mortality or both; such as age-at-diagnosis or sex. On the survival scale, this is formulated as:

$${S}_i\left(\mathrm{t}\right)={S}_i^{\ast }(t){R}_i(t),$$

with *S*_*i*_(t) the all-cause survival function, ${S}_i^{\ast }(t)$ the expected survival function, and *R*_*i*_(*t*) the relative survival function. It is common to report marginal measures in population subgroups or the population as a whole. Much of the recent literature in this research area relates to calculating the appropriate weighting required to arrive at the correct marginal non-parametric estimates for the various quantities of interest.

### Marginal measures

Pohar Perme et al. [2] detail the appropriate weighting that gives an unbiased estimate of the marginal relative survival, and the so-called Pohar Perme estimator is now widely used in practice. Rather than reporting the marginal relative survival, others have recommended the use of crude probabilities; i.e. the partitioning of the all-cause probability of death (*F*(*t*) = 1 − *S*(*t*)) into the probability of death due to cancer (*F*_*C*_(*t*)) and due to other causes (*F*_*O*_(*t*)):

$$F(t)=1-S(t)={F}_C(t)+{F}_O(t)$$

Following a similar notation to Sasieni and Brentnall [25], let *N*_*i*_(*t*) be a counting process that starts at 0 and jumps to 1 at the time when individual *i* dies, and *Y*_*i*_(*t*) be an at risk process – an indicator of whether an individual is still at risk at time *t* (1 if so, 0 otherwise), effectively *Y*_*i*_(*t*) = *I*(*T*_*i*_ ≥ *t*) with *I*() an indicator function. We can define *dN*_*i*_(*t*) = *Y*_*i*_(*t*)*I*(*T*_*i*_ = *t*), which counts the events specifically at time, *t*. We can then sum over all individuals at time *t*; let $dN(t)=\sum_{i=1}^nd{N}_i(t)$ be the sum over all individual events at time *t*, and $Y(t)=\sum_{i=1}^n{Y}_i(t)$ be the total number of individuals at risk at time *t*. Taking a continuous time approach in the non-parametric context; for all individuals *i (i = 1…N)* at risk at time *t*, we can define the marginal all-cause cumulative hazard, $\hat{\mathrm{H}}\left(\mathrm{t}\right),$ as:

1 $$\widehat{\mathrm H}\left(\mathrm t\right)=\int_0^t\frac{dN(s)}{Y(s)}=\int_0^t\frac{\sum_{i=1}^ndN_i(s)}{\sum_{i=1}^nY_i(s)}$$

This can be used in the estimation of the observed all-cause probability of death, $\hat{F}(t)$, above; $\hat{F}(t)=1-\hat{\mathrm{S}}(t)=1-\exp \left(-\hat{\mathrm{H}}(t)\right)$. We can also derive the cumulative expected hazard for each individual using the mortality rate information for the general population, using the mortality rates based on an individual’s, *i*, characteristics at time, *t* (i.e matched on attained age, attained calendar year, and demographic characteristics; such as sex, deprivation group etc.). The cumulative excess hazard for each individual, ${\mathrm{H}}_i^{\ast }(t)$, is estimated up to time, *t*, from the population mortality rate information, ${\mathrm{h}}_i^{\ast }(t):$

$${\mathrm{H}}_i^{\ast }(t)={\int}_0^t{\mathrm{h}}_i^{\ast }(s)\ ds,$$

with the expected (population) survival for each individual given by ${S}_i^{\ast }(t)=\exp \left(-{\mathrm{H}}_i^{\ast }(t)\right).$ There are a number of options for averaging these estimates to arrive at a marginal expected survival for the population and the appropriate approach depends on the context [2, 15].

### Reference adjusted all-cause measures

Our aim is to obtain reference adjusted all-cause survival so that differences are solely due to differences in cancer (excess) mortality. To do this a second group of expected mortality rates needs to be defined, so we can estimate what the all-cause survival would be in a population with the reference expected mortality rates. Lambert et al. [23] recommend the use of a second common set of population mortality rates for the purpose of adjusting to a reference population. With mortality rate information for an individual based on the rates in the second set of population mortality rates denoted with, ${\mathrm{h}}_i^{\ast \ast }(t),$ one can arrive at the population survival under the reference standard population for an individual, *i,* ${S}_i^{\ast \ast }(t)=\exp \left(-{\int}_0^t{\mathrm{h}}_i^{\ast \ast }(t)\ \right)=\exp \left(-{\mathrm{H}}_{\mathrm{i}}^{\ast \ast }(t)\right).$

Adopting a second set of population mortality rates will influence the calculation of the all-cause cumulative hazard. Through the introduction of ${\mathrm{h}}_i^{\ast \ast }(t)$, the overall covariate distribution and hypothetical population at risk at time *t* may differ dependent on the relative difference between ${\mathrm{H}}_i^{\ast \ast }(t)$ and ${\mathrm{H}}_i^{\ast }(t).$ To counteract this, weights can be introduced with the relative contribution depending on the ratio of the two expected survival estimates at time *t* for any given covariate pattern that influences expected mortality – here denoted for each individual *i*. Therefore, defining the weights at each timepoint, *t*, as ${w}_i(t)=\frac{S_i^{\ast \ast }(t)}{S_i^{\ast }(t)}$, we can arrive at a reference-adjusted estimate of the marginal all-cause hazard, *H*_*R*_(t), at time *t*:

2 $$\widehat{H_R}(t)=\int_0^t\frac{\sum_{i=1}^nw_i(s)Y_i(s)\left\{dN_i(s)-dH_i^\ast(s)+dH_i^{\ast\ast}(s)\right\}}{\sum_{i=1}^nw_i(s)Y_i(s)}$$

The formula here adapts the marginal all-cause hazard on the population defined in Eq. (1) above in two ways. Firstly, the { $-d{\mathrm{H}}_{\mathrm{i}}^{\ast }(s)+d{\mathrm{H}}_{\mathrm{i}}^{\ast \ast }(s)$ } term replaces the population hazard assumed to be acting in the population with that of the reference standard. Secondly, the weights, *w*_*i*_(*s*), are used to up or downweight individual events and risktime from those that are over or under-represented in the reference population compared to the observed population at time, *t*, in exactly the same way as described by Sasieni and Brentnall [25] for their relative survival index. It is worth noting a number of features of the relation given in Eq. (2) under specific conditions. When ${\mathrm{H}}_{\mathrm{i}}^{\ast \ast }(t)={\mathrm{H}}_{\mathrm{i}}^{\ast }(t)$ for all *t* and *i* this formula collapses to the Nelson Aalen estimator expressed in Eq. (1); that is with the reference population mortality rates being equivalent to the population expected mortality does not alter the all-cause hazard (nor the consequent calculations of crude probability estimates). Removing the $d{\mathrm{H}}_{\mathrm{i}}^{\ast \ast }(s)$ for all *t*, we arrive at the net cumulative hazard estimator proposed by Sasieni and Brentnall, and this is therefore the all-cause hazard extension of their estimator. Further adaptations of the formula above can also arrive at the Pohar Perme net cumulative hazard (setting ${S}_i^{\ast \ast }(t)=1$), or the Ederer II estimator (setting *w*_*i*_(*t*) = 1), as noted by Sasieni and Brentnall [25].

### Reference adjusted crude probability measures

The reference-adjusted all-cause hazard can then be de-composed to give the reference-adjusted crude probabilities of death due to cancer and other causes using the same weighting, *w*_*i*_(*t*). Defining the marginal net cumulative hazard using the reference standard as $\hat{\Lambda_R}\left(\mathrm{t}\right)$:

$$\widehat{\Lambda_R}\left(\mathrm t\right)=\int_0^t\frac{\sum_{i=1}^nw_i(s)Y_i(s)\left\{dN_i(s)-d\mathrm H_{\mathrm i}^\ast(s)\right\}}{\sum_{i=1}^nw_i(s)Y_i(s)}$$

which is the estimator proposed by Sasieni and Brentnall. And the marginal expected cumulative hazard for the population hazard with the reference standard, $\hat{H_R^{\ast \ast }}\left(\mathrm{t}\right):$

$$\widehat{H_R^{\ast\ast}}\left(\mathrm t\right)=\int_0^t\frac{\sum_{i=1}^nw_i(s)Y_i(s)\left\{d\mathrm H_{\mathrm i}^{\ast\ast}(s)\right\}}{\sum_{i=1}^nw_i(s)Y_i(s)}$$

The relevant crude probability of death due to cancer, ${\mathrm{F}}_R^{\mathrm{C}}\left(\mathrm{t}\right),$ and other causes, ${\mathrm{F}}_R^{\mathrm{O}}\left(\mathrm{t}\right),$ under the reference adjustment can be estimated by:

$$\widehat{\mathrm F_R^{\mathrm C}}\left(\mathrm t\right)=\int_0^t\widehat{S_R}\left(u-\right)d\widehat{\Lambda_{\mathrm R}}(u)$$

$$\widehat{\mathrm F_R^{\mathrm O}}\left(\mathrm t\right)=\int_0^t\widehat{S_R}\left(u-\right)d\widehat{\mathrm H_{\mathrm R}^{\ast\ast}}(u),$$

With *S*_*R*_(*t*) being the all-cause survival function at time, *t*, under the reference standard (estimated as $\hat{S_R}(t)=\exp \left(-\hat{H_R}\left(\mathrm{t}\right)\right)$. We give details of variance estimates and the calculation of confidence intervals implemented in the corresponding software in Appendix A1.

### Standardisation of covariates

Furthermore, it may also be necessary to standardise to a specific covariate pattern (such as an age profile) to allow direct comparability between groups or across different studies. This can be achieved with a modification to the weights, *w*_*i*_(*t*), with a multiplication through by time-fixed weights equivalent to those that have been used in age-standardisation traditionally [25–27], ${w}_i^B$. Redefining the weights as:

$${w}_i(t)={w}_i^B\left(\frac{S_i^{\ast \ast }(t)}{S_i^{\ast }(t)}\right)$$

For instance, these further pre-weights, ${w}_i^B$, have been used for external age-standardisation using the International Cancer Standard Survival weights. In that case, the weights $, {w}_i^B$, correspond to an individual’s age (and the age-group to which they belong), and are a relative comparison of the proportions in each age-group between the current sample and the external standard. Again, the combination of these two sets of weights are also proposed by Sasieni and Brentnall [25], but we convert their standardised relative survival estimates to an all-cause and crude probability setting. The weights can be applied to all of the reference-adjusted measure described above, so that any differences between groups are only due to differences in excess mortality rates.

### Software implementation

In this paper, we use a continuous time implementation of the above approach, with the time-dependent weights, *w*_*i*_(*t*), re-calculated at each unique event time. The software implementation is via a user-written package in Stata; stpp. Further details on the implementation are given in the Appendix (Appendix A2). Cronin and Feuer [24] offer a lifetable approximation (calculation in interval periods, such as months) to continuous time calculation for the observed crude probability estimates. It would be also possible to make similar adjustments to the Cronin and Feuer lifetable approximation approach by applying the reference population mortality information, with the appropriate adjustments for the interval nature of the calculation as introduced by Cronin and Feuer.

### Illustrative example

We select two regions in England to make regional comparisons of survival following rectal cancer for men diagnosed in the calendar period 2007–2012, with follow-up information available until the end of 2013. For the calculation of relative survival, we use a population lifetable stratified by age, deprivation, region, and sex. Given known discrepancies in deprivation-specific relative survival for rectal cancer [28], it is important to quantify if region-specific survival differences remain that are not due to differences in the proportion of men diagnosed in each deprivation group in a given region or due to differences in the age distribution of men diagnosed with rectal cancer across regions. There are also regional variations in other-cause mortality in England [29–31]. We therefore adopt an approach of reference-adjusted and standardised survival comparison. The reference expected mortality rates are for men in England as a whole in 2012. For our standardisation approach we standardise to the age (in 5 broad age-groups; 15–44, 45–54, 55–64, 65–74, 75+) and deprivation distribution of men diagnosed with rectal cancer for England as a whole for the study period of interest. In using a common reference for population mortality rates, and a common covariate distribution for age and deprivation, we provide all-cause and crude probability estimates that offer a fair comparison of the survival impact following a diagnosis of rectal cancer across the government office regions in England. We select two regions with different covariate profiles for illustration; the North East and South East regions. In adopting this approach, we remove the impact of regional variation in other-cause mortality on our estimates, and also any regional variation in the age and deprivation distribution of diagnoses – both of which would otherwise impact on the all-cause and cancer-specific cumulative probabilities of death.

## Results

Table 1 describes the cohort of men diagnosed with rectal cancer between the years 2007 and 2012, and further summarises the age and deprivation distribution separately by the two government office regions. There are substantial differences in the distribution of deprivation across the two regions of England (31% in the most deprived group in North East, compared to 5% in the South West). This likely largely reflects regional variations in the deprivation distributions in the population in each region, rather than differential rectal cancer incidence by deprivation group across the regions. The age profile of the incidence of rectal cancer is relatively similar across the regions. In England as a whole, over 34% of cases of rectal cancer are in those over the age of 75, the oldest age-group we consider.

**Table 1:** Description of cohort for diagnoses of rectal cancer for men diagnosed between 2007 and 2012 in England, and the two comparison regions

**Region**	**Age-group** **N** **(row percentage)**					**Total** **N**
	**15-44**	**45-54**	**55-64**	**65-74**	**75+**	
**North East**	56 (2.2%)	200 (8.0%)	579 (23.1%)	815 (32.5%)	857 (34.2%)	2,507
**South East**	165 (2.6%)	499 (7.7%)	1,420 (22.0%)	2,059 (31.9%)	2,321 (35.9%)	6464
**England** **(TOTAL)**	1,033 (2.5%)	3,181 (7.7%)	9,293 (22.6%)	13,563 (33.0%)	14,051 (34.2%)	41,121
	**Deprivation Group** **N** **(row percentage)**					**Total** **N**
	**1- least ** **deprived**	**2**	**3**	**4**	**5 – most ** **deprived**	
**North East**	356 (14.2%)	346 (13.8%)	440 (17.6%)	594 (23.7%)	771 (30.8%)	2,507
**South East**	2,124 (32.9%)	1,612 (24.9%)	1,432 (22.2%)	965 (14.9%)	331 (5.1%)	6464
**England** **(TOTAL)**	8,479 (20.6%)	9,091 (22.1%)	8,825 (21.5%)	7,910 (19.2%)	6,816 (16.6%)	41,121

Table 2 shows the 5-year probability of all-cause death following a diagnosis of rectal cancer for men in two regions of England. The 5-year crude probability of death due to cancer is also shown. Three estimates are given for each of the above metrics; i) the observed value in each region (i.e. unstandardized and non-reference adjusted); (ii) the impact of the reference adjustment lifetable alone, and iii) the fully standardised and reference adjusted value. In a separate column of Table 2, the fully standardised and reference adjusted net probability index value is given (the Sasieni and Brentnall approach). The observed (unstandardized and non-reference adjusted) values are quite similar across the two compared regions; the all-cause 5-year probabilty of death is 51.1% in the North East region and 50.8% in the South East. However, these values are based on the covariate distributions and other-cause mortality rates of each region separately. Applying the reference-adjustment (all-England rates in 2012), and the covariate standardisation to England as a whole makes a marked difference for these two regions. In the North East region the estimates decrease when reference adjusting and standardising; this is largely driven by the shift in the deprivation distribution (see Table 1, and the column showing reference adjustment alone in Table 2). In contrast, the estimates for the South East increase when using the reference adjustment and standardisation. This is again largely driven by the shift in deprivation distribution, but also by altering to the all-England reference rates for other cause mortality. Comparing the regions when the covariate distribution and other cause mortality have been standardised shows that the North East region has a lower all-cause probability of death than the South East (48.4% vs 52.6% respectively). Table 2 shows also shows the reference adjusted and standardised net probability valeus for each region. These estimates are slightly higher than the reference-adjusted and standardised crude probability of deaths due to cancer because they are net rather than crude estimates that attempt to remove the impact of other-cause mortality that is present in the reference population.

**Table 2:** Regional values for the 5-year reference and standardised marginal probabilities of death (due to cancer and all-causes) following a rectal cancer diagnosis for men (all ages). The reference standard is the population mortality rates for men in England in 2012, and the standardisation group is the joint deprivation and age distribution of men with rectal cancer in England as a whole. Also given for comparison, are values for the observed (unstandardized and non-reference adjusted) probabilities, and the net probability index (Sasieni and Brentnall)

	Crude probability due to cancer			Net probability of death due to cancer	All-cause death probability		
	Observed (unstandardized and non-reference adjusted)	Reference-adjusted only	Reference-adjusted and standardised	Reference-adjusted and standardised (Sasieni & Brentnall)	Observed (unstandardized and non-reference adjusted)	Reference-adjusted only	Reference-adjusted and standardised
North East	39.3%	39.6%	37.3%	39.1%	51.1%	50.4%	48.4%
South East	40.1%	40.0%	41.6%	43.6%	50.8%	51.5%	52.6%

**Fig. 1:**
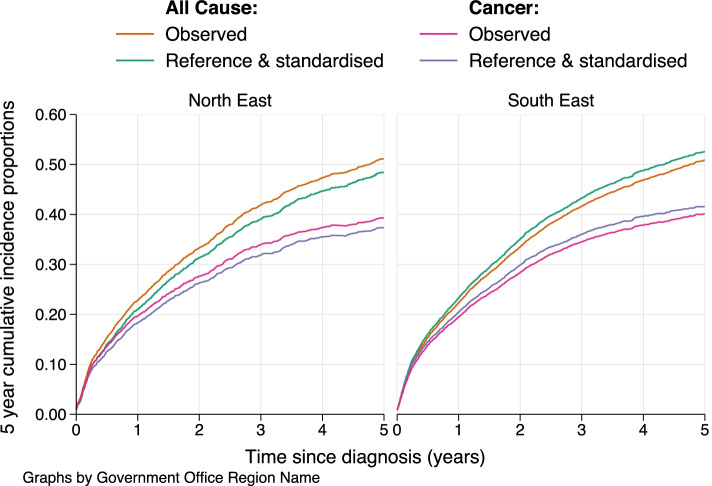
Reference and standardised crude probability estimates (due to cancer and all-causes) for males diagnosed with rectal cancer in two English regions across time since diagnosis (all ages). The reference standard is the population mortality rates for men in England in 2012, and the standardisation group is the joint deprivation and age distribution of men with rectal cancer in England as a whole. Also given, are values for the observed (unstandardized and non-reference adjusted) probabilities

Figure 1 shows the corresponding values across the entire range of time since diagnosis, as opposed to the point estimates given at 5-years in Table 2. The differential impact of reference adjustment for both the all-cause probability of death and the crude probability of death due to cancer can be clearly visualised. For the North East region, the reference adjustment results in a reduction in the probabilities of death; this is because a more favourable deprivation distribution is being applied through the standardisation. In contrast, for the South East, the reference and standardised estimates are higher across the entire range of follow-up than the corresponding observed values.

## Discussion

Cancer survival metrics are typically calculated in the relative survival setting, and often externally age-standardised marginal relative survival will be reported when comparing across regions or countries (or stratifications for population subgroups) in order to remove the effect of differential other-cause mortality. This measure is less relevant for patients and policy makers as it does not reflect the real-world experience of cancer patients, however, it does allow for direct comparability across groups with differential other cause mortality [32]. In this paper, we show that it is possible to maintain this comparability whilst also retaining the overall burden of mortality (from deaths due to cancer and other causes) when reporting metrics, offering an alternative approach to solely reporting net survival metrics, but to do so requires the definition of a reference standard for other cause mortality. The choice of the reference standard is important, but careful choice means that the estimates reported can closely reflect the observed survival experience of the cohort, whilst maintaining direct comparability. In some settings, it may be preferable to report standard net survival measures. One possible example is for large international studies comparing trends in cancer survival across time and between countries. In such studies, the added complexity of choosing a reference standard for other cause mortality might be unnecessary. Net survival measures may also be preferable when the other-cause mortality across the comparison groups are very different. Crucially, the crude probability metrics developed here mean that a broader range of metrics are available that allow direct comparability across population groups and thereby also allowing comparisons tailored to different audiences. Further, these reference adjusted crude probability measures may be better suited in settings when it is important to portray the level of competing mortality.

Net survival metrics are popular in that, under assumptions of conditional exchangeability, these completely remove the impact of other causes by effectively setting this to zero, and calculating the survival function in the scenario where cancer is the only possible cause of death. This is often achieved through estimation in the relative survival framework, where a further assumption of relying on the population mortality rates to be the correct rates due to competing causes for the cancer cohort. Our approach uses this same estimation framework, but rather than assuming that the rate due to competing causes is zero, we adopt a secondary reference population mortality file, which is common across all comparison groups to allow fair real-world comparisons. This maintains comparability, whilst improving the interpretability. Although, the exact interpretation of the measure, is still a complex formulation, it often remains very close to the observed all-cause and cancer-specific probabilities of death for each group of interest. This will be particularly true if the comparison groups of interest are very similar to each other in terms of the rates of other cause mortality and further have similar rates to the chosen the reference standard. In our example, we were able to standardise to all of England (in terms of other-cause mortality rates and the covariate distribution) for regional comparisons across England, which offers a logical choice of common other-cause mortality rate and covariate distribution.

Our approach builds upon the model-based metrics that have been developed by Lambert et al. [23]. We have developed non-parametric equivalents for the same underlying estimands; reference-adjusted and standardised all-cause and cause-specific crude probabilities of deaths. The model-based approach [23] relies on a fully parametric setting; requiring the correct specification of the functional form for non-linear and time-dependent covariate effects in a modelling framework. Our proposed non-parametric estimation approach removes the requirement of the correct model specification and offers an alternative estimation approach that requires fewer assumptions. The non-parametric estimates could also be used in comparison to the model-based estimates to check if the correct functional form and assumptions surrounding the effects of covariate are appropriate.

A further important consideration when comparing across population groups is to ensure that a like-with-like comparison is undertaken for other key covariates that may differ in their distribution, but also impact on cancer survival. An obvious variable to consider is the age profile of the comparison groups, and it is common practice to age-standardise cancer survival estimates to account for this. The equivalent approach to standardisation is required for the reference adjusted metrics introduced in this paper. As with the reference standard for the other-cause mortality rates, a careful choice of covariate standard is needed to both allow direct comparability, whilst also providing survival estimates that are close to those observed in the population groups. Many previous research papers have adopted a common international age standard for cancer survival comparisons. The International Cancer Survival Standard weights are typically a younger profile than the cancer age distribution seen in England. For net survival measures, the difference in age profile between the ICSS age distribution and that observed in England often do not cause a dramatic difference when comparison ICSS-standardised and non-standardised net measures. However, on the all-cause scale, with age variation in both cancer and other-cause mortality, standardising to an age profile that is younger (e.g. the ICSS standard) can have a more marked difference when comparing ICSS age-standardised to non-standardised all-cause metrics. We further perform standardisation for the deprivation distribution in the regional comparisons in Table 2; it is often necessary to consider other key covariates to standardise over depending on the context of the comparison being made.

Although we standardise across the deprivation distribution to allow fair regional comparisons, this should not be seen as accepting the inequities in cancer survival seen across these population groups. This is done on the basis that to allow fair regional comparisons requires case-mix adjustment, which is only necessary due to the inequities in cancer (and other cause) survival across the deprivation groups. Another comparison of interest, that has been used in other studies, is to calculate the hypothetical gains in survival should the cancer-specific inequities in deprivation group survival be removed [33, 34]. Furthermore, we have used regions in England that were formerly referred to as Government Office Regions. These are fairly large population coverage areas (ranging from ~ 2.5million to ~ 9 million individuals in 2019). For smaller geographical areas, there will be much greater variation and a modelling approach that smooths through the survival estimates, and also borrows strength across regions, would offer a better analysis strategy [35, 36].

The non-parametric estimates can be calculated treating time continuously, or using a lifetable approximation by splitting the timescale into pre-defined intervals (e.g. months). Treating time fully continuously can become computationally expensive in large datasets and over long follow-up intervals with many unique event time values as the time-dependent weights need to be continually updated. Applying the grouped time approach reduces computational time in large datasets with little loss of accuracy if the time intervals are sufficiently short. A further motivation for the lifetable approximation is when this becomes necessary because of data availability reasons (e.g. survival information recorded to the nearest month).

We have shown that non-parametric equivalents of standardised and reference adjusted survival estimates [23] can be obtained for cohorts of cancer patients. Using these metrics as a summary measure of the burden of cancer and also for comparisons across groups is an approach that could be adopted rather than, or in addition to, reporting relative survival measures. The comparability ensured by using relative survival (and age standardisation) is maintained, whilst also closely reflecting the observed all cause survival patterns seen for the cohort of cancer patients; which can be broken down into the relevant contributions of deaths due to cancer and other causes.

## Supplementary Information

**Additional file 1.**

## Data Availability

The data that support the findings of this study are available from Public Health England (https://www.gov.uk/government/publications/accessing-public-health-england-data/about-the-phe-odr-and-accessing-data), but restrictions apply to the availability of these data, which were used under license for the current study, and so are not publicly available.

## References

[1] Estève J, Benhamou E, Croasdale M, Raymond L (1990). Relative survival and the estimation of net survival: Elements for further discussion. Stat Med.

[2] Perme MP, Stare J, Estève J (2012). On estimation in relative survival. Biometrics.

[3] Coleman M, Forman D, Bryant H, Butler J, Rachet B, Maringe C, Nur U, Tracey E, Coory M, Hatcher J, McGahan C, Turner D, Marrett L, Gjerstorff M, Johannesen T, Adolfsson J, Lambe M, Lawrence G, Meechan D, Morris E, Middleton R, Steward J, Richards M (2011). Cancer survival in Australia, Canada, Denmark, Norway, Sweden, and the UK, 1995–2007 (the International Cancer Benchmarking Partnership): An analysis of population-based cancer registry data.

[4] De Angelis R, Sant M, Coleman MP, Francisci S, Baili P, Pierannunzio D, Trama A, Visser O, Brenner H, Ardanaz E, Bielska-Lasota M, Engholm G, Nennecke A, Siesling S, Berrino F, Capocaccia R, EUROCARE-5 Working Group (2014). Cancer survival in europe 1999-2007 by country and age: results of EUROCARE–5-a population-based study. Lancet Oncol.

[5] Allemani C, Matsuda T, Di Carlo V, Harewood R, Matz M, Niksic M, Bonaventure A, Valkov M, Johnson CJ, Estève J, Ogunbiyi OJ, Silva GAE, Chen W-Q, Eser S, Engholm G, Stiller CA, Monnereau A, Woods RR, Visser O, Lim GH, Aitken J, Weir HK, Coleman MP, C. W. Group (2018). Global surveillance of trends in cancer survival 2000-14 (concord-3): analysis of individual records for 37,513,025 patients diagnosed with one of 18 cancers from 322 population-based registries in 71 countries. Lancet.

[6] Arnold M, Rutherford MJ, Bardot A, Ferlay J, Andersson TM, Myklebust TÅ, Tervonen H, Thursfield V, Ransom D, Shack L (2019). Progress in cancer survival, mortality, and incidence in seven high-income countries 1995–2014 (icbp survmark-2): a population-based study. Lancet Oncol.

[7] Quaresma M, Coleman MP, Rachet B (2015). 40-year trends in an index of survival for all cancers combined and survival adjusted for age and sex for each cancer in England and Wales, 1971-2011: a population-based study. Lancet.

[8] Araghi M, Arnold M, Rutherford MJ, Guren MG, Cabasag CJ, Bardot A, et al. Colon and rectal cancer survival in seven high-income countries 2010-2014: variation by age and stage at diagnosis (the icbp survmark-2 project). Gut. 2021;70:114–26. https://gut.bmj.com/content/70/1/114.citation-tools. 10.1136/gutjnl-2020-320625 32482683

[9] Exarchakou A, Rachet B, Belot A, Maringe C, Coleman MP. Impact of national cancer policies on cancer survival trends and socioeconomic inequalities in england, 1996-2013: population based study. BMJ. 2018;360:k764. 10.1136/bmj.k764. 10.1136/bmj.k764 PMC5850596 29540358

[10] Stewart SL, Harewood R, Matz M, Rim SH, Sabatino SA, Ward KC, Weir HK (2017). Disparities in ovarian cancer survival in the united states (2001-2009): Findings from the concord-2 study. Cancer.

[11] Andersen PK, Keiding N (2012). Interpretability and importance of functionals in competing risks and multistate models. Stat Med.

[12] Corazziari I, Quinn M, Capocaccia R (2004). Standard cancer patient population for age standardising survival ratios. Eur J Cancer.

[13] Lambert PC, Dickman PW, Nelson CP, Royston P (2010). Estimating the crude probability of death due to cancer and other causes using relative survival models. Stat Med.

[14] Perme MP, Pavlic K. Nonparametric relative survival analysis with the r package relsurv. J Stat Softw. 2018;87(8):1–27. 10.18637/jss.v087.i08.

[15] Belot A, Ndiaye A, Luque-Fernandez M-A, Kipourou D-K, Maringe C, Rubio FJ, Rachet B (2019). Summarizing and communicating on survival data according to the audience: a tutorial on different measures illustrated with population-based cancer registry data. Clin Epidemiol.

[16] Kipourou D-K, Perme MP, Rachet B, Belot A. Direct modeling of the crude probability of cancer death and the number of life years lost due to cancer without the need of cause of death: a pseudo-observation approach in the relative survival setting. Biostatistics. 2020;kxaa017. 10.1093/biostatistics/kxaa017. 10.1093/biostatistics/kxaa017 PMC8759449 32374817

[17] Charvat H, Bossard N, Daubisse L, Binder F, Belot A, Remontet L (2013). Probabilities of dying from cancer and other causes in French cancer patients based on an unbiased estimator of net survival: a study of five common cancers. Cancer Epidemiol.

[18] Andreassen BK, Myklebust T, Haug ES (2017). Crude mortality and loss of life expectancy of patients diagnosed with urothelial carcinoma of the urinary bladder in Norway. Scand J Urol.

[19] Mozumder SI, Dickman PW, Rutherford MJ, Lambert PC (2018). InterPreT cancer survival: A dynamic web interactive prediction cancer survival tool for health-care professionals and cancer epidemiologists. Cancer Epidemiol.

[20] Wong KF, Lambert PC, Mozumder SI, Broggio J, Rutherford MJ (2019). Conditional crude probabilities of death for English cancer patients. Br J Cancer.

[21] Dasgupta P, Aitken JF, Pyke C, Baade PD (2018). Competing mortality risks among women aged 50-79 years when diagnosed with invasive breast cancer, Queensland, 1997-2012. Breast.

[22] Dasgupta P, Cramb S, Kou K, Yu XQ, Baade PD (2019). Temporal trends in net and crude probability of death from cancer and other causes in the Australian population, 1984-2013. Cancer Epidemiol.

[23] Lambert PC, Andersson TM-L, Rutherford MJ, Myklebust TÅ, Møller B. Reference adjusted and standardized all-cause and crude probabilities as an alternative to net survival in population-based cancer studies. Int J Epidemiol. 2020; (in press). 10.1093/ije/dyaa112 32829393

[24] Cronin KA, Feuer EJ (2000). Cumulative cause-specific mortality for cancer patients in the presence of other causes: a crude analogue of relative survival. Stat Med.

[25] Sasieni P, Brentnall AR. On standardized relative survival. Biometrics. 2017:73:473–82. 10.1111/biom.12578. 10.1111/biom.12578 PMC5507182 27554303

[26] Lambert PC, Dickman PW, Rutherford MJ (2015). Comparison of different approaches to estimating age standardized net survival. BMC Med Res Methodol.

[27] Rutherford MJ, Dickman PW, Coviello E, Lambert PC (2020). Estimation of age-standardized net survival, even when age-specific data are sparse. Cancer Epidemiol.

[28] Syriopoulou E, Morris E, Finan PJ, Lambert PC, Rutherford MJ (2019). Understanding the impact of socioeconomic differences in colorectal cancer survival: potential gain in life-years. Br J Cancer.

[29] Woods LM, Rachet B, Riga M, Stone N, Shah A, Coleman MP (2005). Geographical variation in life expectancy at birth in england and wales is largely explained by deprivation. J Epidemiol Community Health.

[30] Walters S, Quaresma M, Coleman MP, Gordon E, Forman D, Rachet B (2011). Geographical variation in cancer survival in England, 1991-2006: an analysis by cancer network. J Epidemiol Community Health.

[31] Office for National Statistics (2019). Cancer survival smoothed life tables.

[32] Rutherford MJ. Care needed in interpretation of cancer survival measures. Lancet. 2015;385(9974):1162–3. https://www.sciencedirect.com/science/article/pii/S0140673614622923?via%3Dihub. 10.1016/S0140-6736(14)62292-3 25479695

[33] Ellis L, Coleman MP, Rachet B (2012). How many deaths would be avoidable if socioeconomic inequalities in cancer survival in England were eliminated? A national population-based study, 1996-2006. Eur J Cancer.

[34] Jansen L, Kanbach J, Finke I, Arndt V, Emrich K, Holleczek B, et al. Estimation of the potentially avoidable excess deaths associated with socioeconomic inequalities in cancer survival in germany. Cancers (Basel). 2021;13(2):357. Published 2021 Jan 19. 10.3390/cancers13020357. 10.3390/cancers13020357 PMC7835812 33478065

[35] Seppä K, Rue H, Hakulinen T, Läärä E, Sillanpää MJ, Pitkäniemi J (2019). Estimating multilevel regional variation in excess mortality of cancer patients using integrated nested laplace approximation. Stat Med.

[36] Seppä K, Malila N, Pitkäniemi J (2020). Variation in cancer survival between hospital districts and within them in Finland. Acta Oncol.

[37] Henson KE, Elliss-Brookes L, Coupland VH, Payne E, Vernon S, Rous B, Rashbass J (2020). Data resource profile: National cancer registration dataset in England. Int J Epidemiol.

