# Supplementary material for: Non-parametric estimation of reference adjusted, standardised probabilities of all-cause death and death due to cancer for population group comparisons
Source: BMC Med Res Methodol. 2022 Jan 6;22:2. doi: 10.1186/s12874-021-01465-w (PMC8740504; doi:10.1186/s12874-021-01465-w)
Supplement: Supplementary file 1 — Additional file 1. [file 12874_2021_1465_MOESM1_ESM.docx]

**Appendix**

**A1: Uncertainty estimation**

We give the formula that are used to calculate the relevant variance estimates for the reference adjusted and standardised crude probability of death due to cancer. The confidence intervals for the estimates are also provided by the stpp package, with further details on the software implementation given in Appendix A2. The confidence intervals provided by the software use the delta method to provide normal-based confidence intervals on either the log scale, or log(-log) scale dependent on the estimand.

The variance estimate for the reference adjusted and standardised crude probability of death due to cancer is an adaptation of the variance estimate calculation given in Equation 8 by Perme & Pavlič[14], with the adaptations relating to the inclusion of the relevant weights (which are squared as constants in the variance term for the final component related to the variance of the referenced adjusted all-cause survival function), and the inclusion of the corresponding reference population mortality rates in all of the terms. Our proposed variance estimate is given by:

$$\hat{VAR}\hat{{(F}_{R}^{C}}\left( t \right))=\int_{0}^{t} \begin{aligned} \left[ \hat{S_{R}}\left( u \right) \right]^{2}\left[ 1-\frac{\left( \hat{F_{R}^{C}}\left( t \right)-\hat{F_{R}^{C}}\left( u \right) \right)}{\hat{S_{R}}\left( u \right)} \right]^{2} \end{aligned}\frac{\sum_{i=1}^{n} w_{i}^{2}\left( s \right)Y_{i}\left( s \right)\left\{ dN_{i}\left( s \right)-dH_{i}^{*}\left( s \right) +dH_{i}^{**}\left( s \right) \right\}}{\left( \sum_{i=1}^{n} w_{i}\left( s \right)Y_{i}\left( s \right) \right)^{2}}$$

An alternative approach to variance estimation could be the use of non-parametric bootstrapping but this can be computationally expensive, and shows good agreement with the estimates given by the above formulation.

**A2: Stata code for analysis**

The estimates presented in this manuscript are implemented in the Stata user-written package stpp, and are a continuous time representation of the estimates – with the relevant weights and the contribution to the estimates calculated at each unique event time.

Definition of selected variables:

dx- date of diagnosis

dexit – event date

sex – indicator variable for male/female

yydx – year of diagnosis

status – all-cause death indicator

patid – patient ID number

extcomp - variable with relative weights to the age/deprivation group distribution in England as a whole.

popmort_region – population mortality file stratified by age, region, sex and calendar year.

popmort_UK – population mortality file stratified by age and sex only for England in 2012.

stpp is a user-written command. Type: findit stpp in Stata.

*ST-SET THE SURVIVAL DATA WITH 5 YEARS FOLLOW-UP**

stset dexit, failure(status==1) exit(time min(dx+`=5*365.24’,mdy(12,31,2013))) origin(dx) id(patid) scale(365.24)

**OBSERVED

stpp R_pp_o using popmort_region, ///

agediag(agediag) ///

datediag(dx) ///

by(region) ///

pmother(sex dep region) ///

allcause(AC_o) ///

crudeprob(CP_o OC_o) ///

deathprob list(5)

**CREATES VARIABLES AC_O CP_O & OC_O – THE OBSERVED ALL-CAUSE, CRUDE PROBABILITY OF DEATH DUE TO CANCER, AND CRUDE PROBABILITY OF DEATH DUE TO OTHER CAUSES RESPECTIVELY.

**STANDARDISED & REFERENCE ADJUSTED

stpp R_pp_sr using popmort_region, ///

agediag(agediag) ///

datediag(dx) ///

by(region) ///

pmother(sex dep region) ///

using2(popmort_UK.dta, ///

pmother2(sex) ///

pmyear2(.)) ///

allcause(AC_sr) ///

crudeprob(CP_sr OC_sr) ///

deathprob ///

indwei(extcomp) list(5)

**CREATES VARIABLES AC_sr CP_sr & OC_sr – THE STANDARDISED (VIA EXTCOMP) & REFERENCE-ADJUSTED (VIA using2() OPTION) ALL-CAUSE, CRUDE PROBABILITY OF DEATH DUE TO CANCER, AND CRUDE PROBABILITY OF DEATH DUE TO OTHER CAUSES RESPECTIVELY – THE ASSOCIATED UPPER AND LOWER 95% CONFIDENCE INTERVAL VALUES ARE ALSO CREATED IN ADDITIONAL VARIABLES (e.g. CP_sr_lci and CP_sr_uci).
